# Thickness of the Deltoid Muscle and Location of the Anterior Branch of the Axillary Nerve and the Posterior Circumflex Humeral Artery for Deltoid Injections

**DOI:** 10.1155/2022/1784572

**Published:** 2022-12-15

**Authors:** Ye-Gyung Kim, Yoon-Hee Chung, Hee-Jun Ahn, Anna Jeon, Yi-Suk Kim, Kun Hwang, Seung-Ho Han

**Affiliations:** ^1^Department of Anatomy, College of Medicine, Chung-Ang University, Seoul, Republic of Korea; ^2^Department of Anatomy, Catholic Institute for Applied Anatomy, College of Medicine, The Catholic University of Korea, Seoul, Republic of Korea; ^3^Department of Plastic Surgery, Inha University School of Medicine, Incheon, Republic of Korea; ^4^Department of Anatomy, College of Medicine, Ewha Womans University, Seoul, Republic of Korea

## Abstract

This study investigated the thickness of the deltoid muscle and the location of the anterior branch of the axillary nerve (AAN) and posterior circumflex humeral artery (PCHA), with the goal of maximizing the effectiveness of deltoid injections. Forty specimens from 22 adult Korean cadavers were used. A reference line was identified, connecting the anterior point of the deltoid muscle (AP) and the posterior point of the deltoid muscle (PP) on the surface. The midpoint between the AP and PP was used as the origin point (OP). The line connecting the OP and the lowest point of the deltoid tuberosity (DP) was used as the *y*-axis. The mean distance of the reference line from the AP to PP was 4.7 ± 0.7 cm. The vertical mean length of the deltoid muscle from the OP and DP was 16.1 ± 1.0 cm. At the 3, 5, and 7 cm sites, the thickness of the deltoid muscle was 0.62 ± 0.9, 0.73 ± 0.7, and 1.3 ± 1.1 cm, respectively. Most of the branches of the axillary nerve were concentrated in the third section (4-6 cm, 51%), while the branches of the PCHA were predominantly found in the fourth section (6-8 cm, 69%). The peripheral branches of the AAN entering the muscle were distributed between 2.2 and 9.8 cm from the acromion. The mean number of the peripheral branches of the AAN was 9.6 ± 3.4. In the deltoid muscle, the mean number of peripheral branches of the PCHA was 8.2 ± 2.8. Administering deltoid injections 5-6 cm below the OP is recommended to avoid axillary nerve injury.

## 1. Introduction

Deltoid intramuscular injections are a common procedure [1–3]. The mass of the deltoid muscle is smaller than that of other intramuscular injection sites such as the gluteus maximus or the vastus lateralis muscle [4].

To identify the proper location for injection, it is necessary to understand the anatomy of the neurovascular structures beneath the muscle. In particular, to avoid errors in injections into the deltoid muscle, it is necessary to accurately identify the location of the anterior branch of the axillary nerve and PHCA, as well as the thickness of the deltoid muscle itself [5–7].

Anatomical studies of the deltoid muscle have examined the size and shape of the muscle belly and the location of the axillary nerve, and studies on botulinum toxin (BTX) injection have also been conducted [8–14]. However, there are few studies on the relative distribution of the arteries and nerves in the deltoid muscle or the thickness of the deltoid muscle relating to deltoid injections [15, 16].

The aim of this study was to determine the thickness of the deltoid muscle and to identify the location of the anterior branch of the axillary nerve and PHCA, in order to optimize deltoid injections.

## 2. Materials and Methods

### 2.1. Materials

Forty specimens from Korean adult embalmed and nonembalmed cadavers (11 male, 11 female; 10 embalmed, 12 nonembalmed; age range: 49 to 93 years; mean age: 76 years) were used in this study. Specimens without a history of shoulder surgery or fractures were chosen. After removing the skin and subcutaneous tissue overlying the deltoid muscle, a detailed dissection was performed in all specimens, while taking extreme care to avoid damaging the axillary nerve. In this study, we focused on the acromial part of the deltoid muscle.

### 2.2. Landmarks and Axis

**Figure 1:**
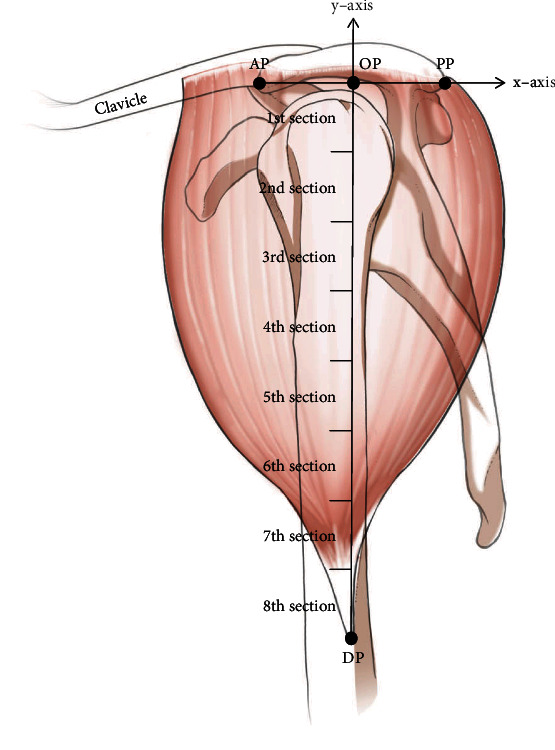
Illustration of the reference lines used to measure the thickness of the deltoid muscle and length of the bony landmark. The line connecting the OP and DP was equally divided into 8 equal sections. The average value of each section was about 2 cm. AP: the anterior point of the acromion; PP: the posterior point of the acromion; OP: the origin point of the reference line; blue line: thickness measurement point of the deltoid muscle; *x*-axis: line connecting the AP and PP; DP: the lowest point of the deltoid tuberosity; *y*-axis: line connecting the OP and DP.

The anterior point of the acromion (AP), the posterior point of the acromion (PP), and the acromion were identified before dissection. The origin point (OP) was defined as the midpoint of a reference line (*x*-axis) connecting the AP and PP at the acromion level, and a vertical line (*y*-axis) was formed by connecting the OP to the lowest point of the deltoid tuberosity (DP) (Figure 1).

### 2.3. Methods of Measuring the Thickness of the Deltoid Muscle, Skin, and Soft Tissue

The thickness of the deltoid muscle, skin, and soft tissue was measured using digital calipers (resolution 0.01 mm, CD-20PSX, Mitutoyo, Japan) at the 3, 5, and 7 cm distance from the OP.

### 2.4. Location of the Anterior Branch of the Axillary Nerve and the Posterior Circumflex Humeral Artery

The distribution of the branches of the axillary nerve and the PCHA was investigated to determine how many branches were located in each area, after dividing the branches into eight equal sections on the *y*-axis. Since the anterior branch of the axillary nerve is the main nerve in the acromial part of the deltoid muscle, we excluded the posterior branch of the axillary nerve from our study and focused only on the anterior branch of the axillary nerve.

### 2.5. Statistical Analysis

Data were analyzed using Microsoft Excel (Excel 2016, Microsoft Corp., Redmond, WA, USA). The *t*-test was used to compare the variables of interest according to sex, and *P* values less than 0.05 were considered to indicate statistical significance. The present study was conducted in accordance with the principles of the Declaration of Helsinki. This study was approved by the Institutional Review Board of Chung-Ang University (IRB No: 1041078-201903-BRBM-089-01).

## 3. Results

### 3.1. Landmarks and the Muscle

The mean length of the reference line from the AP to PP was 4.7 ± 0.7 cm (maximum, 6.4 cm; minimum, 3.2 cm). The mean vertical length of the deltoid muscle from the OP and DP was 16.1 ± 1.0 cm (maximum, 18.3 cm; minimum, 14.7 cm) (Table 1).

The thickness of the deltoid muscle was 0.67 ± 13 cm(range, 0.51–0.84 cm), 0.79 ± 0.12 cm (range, 0.64–0.96 cm), and 1.32 ± 1.0 cm (range, 1.15–1.40 cm) at 3, 5, and 7 cm above the *x*-axis, respectively (Table 2).

### 3.2. Location of the Anterior Branch of the Axillary Nerve and the PCHA

The main branches of the axillary nerve and the PCHA were located in the transverse area of the surgical neck of the humerus at the deltoid muscle. To analyze the distribution of the nerve and artery, we divided the region into eight horizontal compartments, with 2 cm intervals on the *y*-axis (from the OP to the DP) (Figure 1).

**Figure 2:**
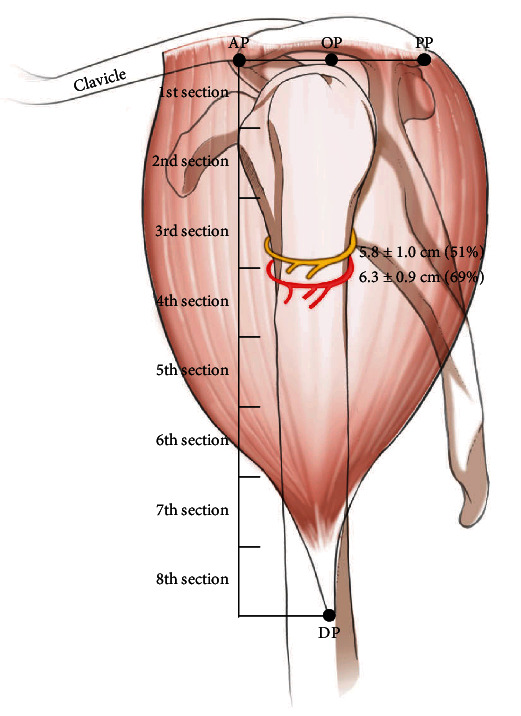
The distribution location of the main branches of the axillary nerve and the PCHA in the deltoid muscle. The axillary nerve and PCHA were located at a mean distance of 5.8 ± 1.0 cm and 6.3 ± 0.9 cm downward from the AP-PP line (*x*-axis), respectively. Most of the main branches of the axillary nerve were concentrated in the third section (4-6 cm, 51%), with the branches of the PCHA in the fourth section (6-8 cm, 69%).

The axillary nerve and the PCHA were distributed at 5.8 ± 1.0 cm and 6.3 ± 0.9 cm from the *x*-axis, respectively. Most of the main branches of the axillary nerve (observed during macroscopic investigations) were concentrated in the third section (4-6 cm, 51%), with the branches of the PCHA in the fourth section (6-8 cm, 69%) (Table 3 and Figure 2). The PCHA ran 0.5 cm below the axillary nerve on average, with no statistically significant difference by sex (*P* = 0.554, *t*-test).

### 3.3. Entry Points of the Peripheral Branches of the Axillary Nerve and the PCHA

The peripheral branches of the axillary nerve, which entered the muscle, were distributed between 2.2 and 9.8 cm from the acromion. The mean number of peripheral branches of the axillary nerve was 9.6 ± 3.4, and the peripheral branches of the axillary nerve entering the deltoid muscle were distributed as follows: ≤5 branches: 5 specimens (17%); 6 to 10 branches: 11 specimens (37%); and 11 to 15 branches: 14 specimens (46%).

The number of the PCHA branches entering the deltoid muscle ranged from 2 to 13. In the deltoid muscle, the mean number of peripheral branches of the PCHA was 8.2 ± 2.8, and the branches entering the deltoid muscle were distributed as follows: ≤5 branches: 4 specimens (15%); 6 to 10 branches: 17 specimens (65%); and 11 to 15 branches: 5 specimens (20%).

**Figure 3:**
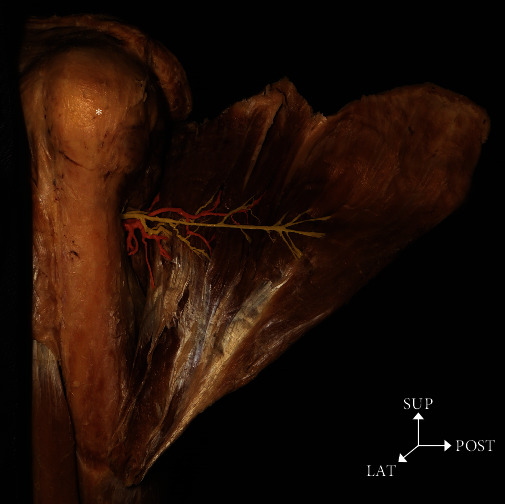
A photograph showing the axillary nerve (yellow color) and the PCHA (red color) distributed in the medial part by dissecting the deltoid muscle on the left side. Asterisk: head of the humerus; SUP: superior; LAT: lateral.

The number of peripheral branches of the axillary nerve was most often between 11 and 15, and the number of peripheral branches of the PCHA was most often between 6 and 10 (Figure 3 and Table 4).

### 3.4. The Main Branching Pattern of the PCHA

The PCHA passed through the deltoid muscle after bifurcating into one branch or two branches. The pattern bifurcating into two branches was the most common (type A, 60%). In types B and C, it was divided into inferior (type B, 35%) and superior (type C, 5%) branches, respectively.

**Figure 4:**
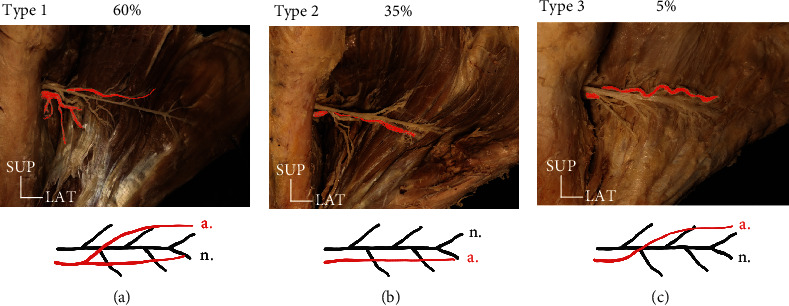
Photographs and schematic illustrations indicating the distribution patterns of the main branch of the PCHA. (a) Type A (the two branches), (b) Type B (the inferior one branch), (c) Type C (the superior one branch). Red color: PCHA; SUP: superior; LAT: lateral.

It was found that the main trunk of the PCHA was distributed below the axillary nerve in all samples (Figure 4).

## 4. Discussion

The deltoid muscle is widely used for intramuscular injections and as an injection point in the fields of anesthesia and vaccination [2, 3, 17, 18], as well as in rehabilitation medicine for injections to treat shoulder pain or muscle sprains [19]. From our observations, the axillary nerve and PCHA distributed in the deltoid muscle ran closely to each other along the surgical neck of the humerus, as two structures were about 0.5 cm apart. In our study, the positional relationship between the axillary nerve and the PCHA was studied through cadaveric dissection to identify effective injection points in the deltoid muscle.

In our study, there was a significant difference in the length between the AP and PP, which is a bony landmark between males and females (*P* = 0.002, *t*-test). Nicholson et al. also reported that there was a significant difference in the length of the acromion between males and females, and multiple regression analysis revealed no significant changes in any dimension with increasing age. Our results agree with those of the study by Nicholson et al. [20].

A study by Nakajima et al. described the location of the nerves and arteries distributed in the deltoid muscle and reported a safe point for intramuscular injection [2]. They recommended that the safest point for deltoid muscle injections was the periphery of the deltoid tuberosity (about 9.9-12.0 cm), which is located far away from the nerves and arteries. In contrast, in the present study, the axillary nerve and arteries were mainly distributed in the third (51%) and fourth (45%) sections, respectively. Therefore, for safe injections into the deltoid muscle, one must inject along the axillary nerve in a horizontal direction from about 5–6 cm below the *x*-axis along the surface of the humerus. The deltoid muscle and skin are thickest at the 7 cm point. We suggest that deltoid muscle injections could be performed safely unless the injection is performed too deeply. In deltoid muscle contouring, a small dose should be spread out and injected widely into the superficial layer of the muscle in consideration of the spread of BTX, and care should be taken not to damage the arteries. Anatomical variations are commonly found and are statistically predictable, and multiple observations can help to overcome the researcher subjectivity [21, 22].

In comparison with the Nakajima et al.'s study, the distribution location of the PCHA in males was different from our study. In Nakajima et al.'s study and in our study, the PCHA in males were 7.6 ± 1.0 cm and 6.5 ± 1.0 cm below the midacromion, respectively. We observed that the PCHA in male is present below in Nakajima et al.'s study. In Nakajima et al.'s study, the length of the PCHA in the male was significantly longer (*P* ≤ 0.001, *t*-test). However, no significant difference was found between the Korean and the Japanese in humerus length (*P* = 0.449, *t*-test) [23, 24]. The difference in the length of the deltoid muscle, even though there was no difference in the humerus, identified that there was a difference between the two races regardless of the humerus.

In this study, the distribution pattern varied according to the specimen. The location of the entry point of the peripheral branch that entered the muscle was between 2.2 and 9.8 cm below the *x*-axis (AP-PP line). In particular, the fourth section (69%) should be avoided for injections because the main branches of the PCHA are distributed in that region. The locations of the main branches of the axillary nerve and the PCHA were found to be 5.8 ± 1.0 cm and 6.3 ± 0.9 cm away from the AP-PP line, respectively. From the AP and PP lines, the main branch of the PCHA was located at 6.3 ± 0.9 cm, and the peripheral branches were located at 8.2 ± 2.8 cm, mainly distributed in sections 4 and 5 in the entire deltoid muscle.

The findings of this study have important implications for maximizing the effectiveness of injections and reducing complications. If Sihler's staining of the deltoid muscle is performed in a follow-up study, it will be possible to resolve the limitations of macroscopic anatomy by revealing the innervation of the muscle. The identification of the exact location of the distribution patterns of nerves and arteries in the deltoid muscle is very important for injections into the deltoid muscle; hence, the information obtained in this study is of value.

## Figures and Tables

**Table 1:** Mean values of the reference line measurements (unit: cm).

Measurements	Total (mean ± SD)	Males (mean ± SD)	Females (mean ± SD)	*P*
AP-PP length	4.7 ± 0.7	5.0 ± 0.7	4.5 ± 0.7	0.002
OP-DP length	16.1 ± 1.0	16.3 ± 1.1	16.0 ± 0.9	0.517

SD: standard deviation; AP: the anterior point of the acromion; DP: the lowest point of the deltoid tuberosity; PP: the posterior point of the acromion; OP: the origin point of the reference line (*x*-axis).

**Table 2:** Measurements of the thickness of the deltoid muscle (on the *y*-axis) (unit: cm).

	3 cm	5 cm	7 cm
	Mean (range)		
Deltoid muscle thickness	0.67 (0.51–0.84)	0.79 (0.64–0.96)	1.32 (1.15–1.40)

SD: standard deviation.

**Table 3:** Locations of the main branches of the axillary nerve and the PCHA in relation to the OP (unit: cm).

Measurements	Total (mean ± SD)	Males (mean ± SD)	Females (mean ± SD)	Min	Max	*P*
Axillary nerve	5.8 ± 1.0	6.0 ± 1.0	5.7 ± 0.9	4.5	8.5	0.107
Posterior circumflex humeral artery	6.3 ± 0.9	6.5 ± 1.0	6.2 ± 1.0	4.0	8.3	0.554

SD: standard deviation.

**Table 4:** The number of peripheral branches of the axillary nerve and the PCHA entering the deltoid muscle (unit: cm).

	Axillary nerve	Posterior circumflex humeral artery
Mean ± SD	9.6 ± 3.4	8.2 ± 2.8
0-5 (%)	5 (17)	4 (15)
6-10 (%)	11 (37)	17 (65)
11-15 (%)	14 (46)	5 (20)

SD: standard deviation.

## Data Availability

The underlying data supporting the results of our study can be found in the manuscript.

## References

[1] Lippert W. C., Wall E. J. (2008). Optimal intramuscular needle-penetration depth. *Pediatrics*.

[2] Nakajima Y., Mukai K., Takaoka K. (2017). Establishing a new appropriate intramuscular injection site in the deltoid muscle. *Human Vaccines & Immunotherapeutics*.

[3] Malkin B. (2008). Are techniques used for intramuscular injection based on research evidence. *Nursing Times*.

[4] Hwang K., Nam Y. S., Han S. H., Hwang S. W. (2009). The intramuscular course of the inferior gluteal nerve in the gluteus maximus muscle and augmentation gluteoplasty. *Annals of Plastic Surgery*.

[5] Orellana-Donoso M., Valenzuela-Fuenzalida J. J., Gold-Semmler M., Guernica-Garcia-Gorigoitia, Shane-Tubbs R., Santana-Machuca E. (2021). Neural entrapments associated with musculoskeletal anatomical variations of the upper limb: literature review. *Translational Research in Anatomy*.

[6] Bergman R. A., Afifi A. K., Miyauchi R. (2015). Illustrated Encyclopedia of Human Anatomic Variation: Opus II: Cardiovascular System: Arteries: Upper Limb.

[7] Clarke E., Mazurek A., Radek M. (2021). Superficial brachial artery - a case report with commentaries on the classification. *Translational Research in Anatomy*.

[8] Kontakis G. M., Steriopoulos K., Damilakis J., Michalodimitrakis E. (1999). The position of the axillary nerve in the deltoid muscle: a cadaveric study. *Acta Orthopaedica Scandinavica*.

[9] Moatshe G., Marchetti D. C., Chahla J. (2018). Qualitative and quantitative anatomy of the proximal humerus muscle attachments and the axillary nerve: a cadaveric study. *Arthroscopy: The Journal of Arthroscopic & Related Surgery*.

[10] Loukas M., Grabska J., Tubbs R. S., Apaydin N., Jordan R. (2009). Mapping the axillary nerve within the deltoid muscle. *Surgical and Radiologic Anatomy*.

[11] Cetik O., Uslu M., Acar H. I., Comert A., Tekdemir I., Cift H. (2006). Is there a safe area for the axillary nerve in the deltoid muscle?. *The Journal of Bone & Joint Surgery*.

[12] Gurushantappa P. K., Kuppasad S. (2015). Anatomy of axillary nerve and its clinical importance: a cadaveric study. *Journal of Clinical and Diagnostic Research*.

[13] Seo K. K. (2017). Body contouring with botulinum toxin. *Botulinum Toxin for Asians*.

[14] Shin S. H., Park S. J., Yeoum S. H., Youn C. S., Park K. Y. (2021). Efficacy and safety of botulinum toxin injection in reducing deltoid muscle hypertrophy. *Dermatologic Therapy*.

[15] Wysiadecki G., Polguj M., Krasucki K. (2014). Morphology and a proposed model of innervation of the human deltoid muscle: a pilot study. *Folia Morphologica*.

[16] Leechavengvongs S., Teerawutthichaikit T., Witoonchart K. (2015). Surgical anatomy of the axillary nerve branches to the deltoid muscle. *Clinical Anatomy*.

[17] Davidson K. M., Rourke L. (2013). Teaching best-evidence: Deltoid intramuscular injection technique. *Journal of Nursing Education and Practice*.

[18] Fujimoto E. (2008). The problem of using deltoid muscle for intramuscular injection. *Aino Journal*.

[19] Yang K. H. (2005). Helical plate fixation for treatment of comminuted fractures of the proximal and middle one-third of the humerus. *Injury*.

[20] Nicholson G. P., Goodman D. A., Flatow E. L., Bigliani L. U. (1996). The acromion: morphologic condition and age-related changes. A study of 420 scapulas. *Journal of Shoulder and Elbow Surgery*.

[21] Żytkowski A., Tubbs R. S., Iwanaga J., Clarke E., Polguj M., Wysiadecki G. (2021). Anatomical normality and variability: historical perspective and methodological considerations. *Translational Research in Anatomy*.

[22] Haładaj R., Wysiadecki G., Clarke E., Polguj M., Topol M. (2019). Anatomical variations of the pectoralis major muscle: notes on their impact on pectoral nerve innervation patterns and discussion on their clinical relevance. *BioMed Research International*.

[23] Hong E., Kwak D. S., Kim I. B. (2021). Morphometric evaluation of detailed asymmetry for the proximal humerus in Korean population. *Symmetry*.

[24] Hasegawa I., Uenishi K., Fukunaga T., Kimura R., Osawa M. (2009). Stature estimation formulae from radiographically determined limb bone length in a modern Japanese population. *Legal Medicine*.

[25] Iwanaga J., Singh V., Ohtsuka A. (2021). Acknowledging the use of human cadaveric tissues in research papers: recommendations from anatomical journal editors. *Clinical Anatomy*.

